# The Interplay between the Host Microbiome and Pathogenic Viral Infections

**DOI:** 10.1128/mBio.02496-21

**Published:** 2021-11-02

**Authors:** Rubén González, Santiago F. Elena

**Affiliations:** a Instituto de Biología Integrativa de Sistemas, Consejo Superior de Investigaciones Científicas-Universitat de València, Paterna, Valencia, Spain; b The Santa Fe Institute, Santa Fe, New Mexico, USA; Albert Einstein College of Medicine

**Keywords:** antiviral treatments, host-microbiome interactions, host-virus interactions, interferon, immune modulation, pathogenesis

## Abstract

The microorganisms associated with an organism, the microbiome, have a strong and wide impact in their host biology. In particular, the microbiome modulates both the host defense responses and immunity, thus influencing the fate of infections by pathogens. Indeed, this immune modulation and/or interaction with pathogenic viruses can be essential to define the outcome of viral infections. Understanding the interplay between the microbiome and pathogenic viruses opens future venues to fight viral infections and enhance the efficacy of antiviral therapies. An increasing number of researchers are focusing on microbiome-virus interactions, studying diverse combinations of microbial communities, hosts, and pathogenic viruses. Here, we aim to review these studies, providing an integrative overview of the microbiome impact on viral infection across different pathosystems.

## INTRODUCTION

In 1676, Antonie van Leeuwenhoek saw and described microbes for the first time ever. Still, the study of microorganisms did not advance much until the late 1800s, when Robert Koch and Louis Pasteur pointed at microbes as the cause of transmissible diseases. Since then, microbes had only been associated with the negative impacts they exerted on their host. This view started to change with the rise of the high-throughput sequencing techniques during the past decades and the study of the metagenome of microbial communities. Nowadays we know that all multicellular organisms have an associated microbiota: a set of microorganisms living within/over them and in their immediate surroundings. The term microbiome can allude to the combined genetic material of the microbiota or refer to the microbiota and their theater of activity: microbial structures, metabolites, and their mobile genetic elements (1).

The microbiome is an important factor contributing to the hosts’ health (2). An adequate microbiome is essential not only to keep the organism healthy but also for protecting it from other pathogens. When considering viral infections, the microbiome influences and is influenced by pathogenic viruses. These interactions can affect viral replication, transmission, and the severity of disease (3). The importance of the role the microbiome plays may vary depending on the host’s circumstances. For example, the impact of the microbiome on providing a healthy status to the host would be especially relevant if external factors reduce the host’s defenses (4). Importantly, the interplay between the microbiome and viral infections depends on the microbiome’s species composition and diversity. This microbial composition is dynamic, changing over time depending on multiple factors: the host species (5) and developmental stage (6), aging (7, 8), the particular organ within the host (9, 10), the host immunity (11–13), diet (14, 15), geography (8, 16), infections with other pathogens (17, 18), the host metabolic signaling pathways (19), or even circadian rhythms (20). The microbe’s composition can also be altered due to microbial transfers between humans, other animals, and the environment (21).

When considering the effects of the microbiome, it is important to be aware of the diversity of taxonomical groups that it might contain: bacteria, archaea, algae, viruses, fungus, and other microeukaryotes. The compositions of these microbial communities and their interactions are being studied in diverse organisms: from Homo sapiens (22), Mus musculus (23), and Caenorhabditis elegans (24) to Arabidopsis thaliana (25). Bacteria represent the most abundant microbial community in the microbiome. Thus, not surprisingly, the bacteriome has been the best-studied component of the microbiome. However, the role of other species in the microbial community should not be neglected: the impact of a microbe is not proportional to its abundance in the microbial community. For example, the fungal microbiota, the mycobiome, is starting to be considered an important component of many illnesses, having an influential role in immune responses (26, 27). Other microbes, such as the algae, are also being considered members of the plant microbiome, as they seem to have important functions for their host and are ubiquitous in plant tissues and in their immediate soil surroundings (28). One of the components of the microbial community that has received less attention are viruses. However, a growing number of metatranscriptomic studies have identified enormous viral diversity and interaction within the microbiome (29–31). The virus component of the microbiome, the virome, includes both the host’s endogenous retroviruses, viruses infecting host cells (persistently or acutely), and viruses infecting components of the microbiome (32). The virome can play relevant roles for the host, as it can modulate the immune system (33) or develop the same functions as an entire community of bacteria: e.g., a murine norovirus has the capacity to support intestinal homeostasis and shape mucosal immunity like commensal bacteria (34).

**FIG 1:**
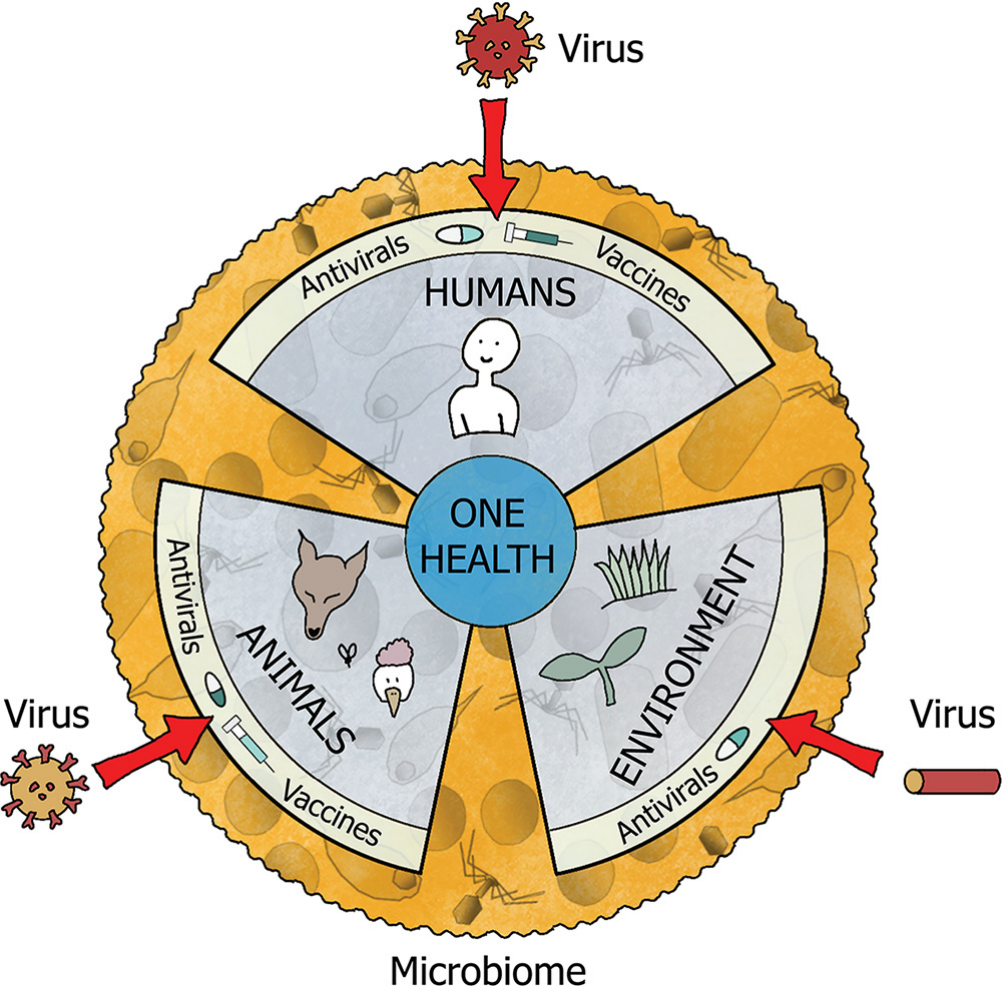
The “One Health” approach seeks an optimal health status for people, animals, and the environment. This goal is constantly threatened by existing and emerging viruses. The microbiome can be a decisive factor to prevent and mitigate the impact of pathogenic viruses. Furthermore, the microbiome can also enhance the effect of the current treatments available against viruses.

This review focuses in a particular role played by the microbiome: the modulation of the host’s viral infections. Even though most viruses are not pathogenic (35), the small fraction of pathogenic ones can cause severe diseases. Pathogenic viruses are also responsible for big economic losses; they have a large negative impact on natural and agricultural ecosystems. For these reasons, studying viral infections while considering the microbial diversity of organisms is highly relevant: integrated approaches that protect both humans, other animals, and the environment in the fight against viruses should be implemented. This approach is commonly known as “One Health” (https://www.cdc.gov/onehealth/). The microbiome is a factor to consider to successfully achieve a common optimal health (Fig. 1): the infectivity, symptomatology, and transmissibility of a virus can be influenced by its host’s microbiome. Furthermore, the microbiome can also alter the efficacy of antiviral therapies. A growing body of research is describing these phenomena by studying the microbial correlations with infection phenotypes and/or the consequences of microbiota manipulation. In the next sections, we will review this research and explore (i) how the microbiome can mitigate or enhance viral infections, (ii) the impact of the microbiome on antiviral treatment and vaccine efficacy, and (iii) future directions in the study of the microbiome-pathogenic virus interactions.

## MICROBIOME MITIGATION OF VIRAL INFECTION

### Humans and other animals.

The current COVID-19 pandemic, caused by severe acute respiratory syndrome coronavirus 2, has increased the awareness of the destructive potential that novel emerging pathogenic viruses might have. Nevertheless, the danger of pathogenic viruses has been always there. Viruses cause millions of deaths in the human population yearly as they are the cause of respiratory and diarrheal diseases, 15% of cancers, and AIDS (36, 37). Viruses affecting farm and wild animals not only can cause ecological and economical losses but also are potentially dangerous to humans, as eventually they can eventually be transmitted to them and originate a zoonotic disease. Therefore, the mitigation of viral infections will reduce disease and death, decrease perturbations of ecosystems, improve the well-being of other species, and boost the economy.

The microbiome might contribute to virus mitigation by enhancing host immunity and reducing the rates of virus replication and infectivity. Kim et al. (38) studied the impact of Staphylococcus epidermidis, a common human nasal commensal, on influenza A virus (IAV) infections. Mice exposed to S. epidermidis suppressed the replication of IAV in the nasal mucosa. This suppression prevented IAV spread to the lung and was caused by the stimulation of interferon (IFN) innate immunity. The same pathway was also activated by S. epidermidis in human cells (38). When facing respiratory syncytial virus (RSV), mice supplemented with *Lactobacillus* showed an enhanced immune response (39). The oral consumption of Lactobacillus paracasei improved the pulmonary immune defense of mice, resulting in reduced susceptibility to RSV infection and faster viral clearance. This effect was partially reproduced when peptidoglycans, a component of the bacterial cell wall, were administered to mice (40). Ji et al. (41) provided neonatal mice with a set of bacteria (Escherichia coli, Streptococcus thermophilus, *Bifidobacterium* spp., and *Lactobacillus* spp.) that suppressed infection with RSV, protecting the host against the lung disease caused by the virus. This defense response was associated with production of IFN-β in alveolar macrophages and the abundance of *Corynebacterium* and *Lactobacillus* in the lungs. Stefan et al. (42) showed that mice colonized with *Bacteroides* spp. can induce IFN-β through Toll-like receptor 4 (TLR4) signaling, which enhances resistance to vesicular stomatitis virus Indiana and IAV. The microbiome can also help in restoring host immunity after perturbation with chronic viral infections. Rhesus macaques chronically infected with simian immunodeficiency virus (SIV) showed an increased immune response when their microbiome composition was changed after a fecal microbiota transplant (43).

The impact of the microbiome on pathogenic viruses can also be tested by studying infection of hosts whose microbiome has been depleted with drugs (axenic). Bradley et al. (44) studied the response of axenic mice to IAV, finding that the microbiota drives an IFN response in the lungs that stops early IAV replication. Yitbarek et al. (45) observed that axenic chickens showed a reduction of their immune response and higher virus shedding after infection with IAV. When axenic chickens were supplemented with a combination of five *Lactobacillus* spp., their immune response was restored and their virus shedding reduced. Figueroa et al. (46) studied ducks infected with the highly pathogenic IAV strain H5N9. They observed that axenic ducks had an increased viral replication and a reduction of the antiviral immune response in the intestine. Yang et al. (47) showed that the mouse microbiome was necessary for protection against encephalomyocarditis virus: axenic mice had reduced mononuclear phagocyte activation and type I IFN responses, which resulted in increased mortality and neuropathogenesis for the host. Providing the axenic mice with a single bacterial microbiome (Blautia coccoides) restored the macrophage activation and type I IFN responses, diminishing virus replication. For chikungunya virus (CHIKV) infections, both germfree and microbiome-depleted mice show an increased viral burden. Providing those mice with Clostridium scindens reduced the viremia due to a restoration of type I IFN responses (48).

Importantly, the microbiome not only could bolster the host response against a pathogenic virus, but also could reduce the severity of the disease. Drosophila melanogaster flies have a rapid mortality when infected with the RNA viruses cricket paralysis virus, *Drosophila* C virus, and Flock House virus. However, Hedges et al. (49) found that this mortality was significantly delayed and reduced when the flies hosted the bacteria Wolbachia pipientis. Wang et al. (50) observed that Staphylococcus aureus, which commonly colonizes the upper respiratory mucosa, significantly attenuates IAV-mediated lung immune injury. Pigs with highly diverse microbiomes or hosting nonpathogenic E. coli strains have an improved outcome after being infected with the porcine reproductive and respiratory syndrome virus (PRRSV) and porcine circovirus type 2 (PCV2) (51). Patin et al. (52) studied the microbiomes of humans and then challenged them with norovirus. They compared the prechallenge microbiome of people with symptoms of virus infection against that of people without symptoms. They found that asymptomatic individuals had microbiomes enriched with *Bacteroidetes* spp. and depleted in *Clostridia* spp. In an experiment with microbiome-depleted hosts, Yaron et al. (53) inoculated axenic mice with murine gammaherpesvirus 68 (MHV-68). These mice had a lower survival rate than the control group. Together, all of these studies exemplify how the microbiome can contribute to the mitigation of the severity of viral infection and even reduce its mortality.

### Vectors of viruses.

Arboviruses are transmitted from one host species to another with the help of vectors. The vectors’ microbiome may alter their competence for acquiring and transmitting viruses. This not only will reduce the infection intensity in the vector population, but also will minimize the incidence of the virus on the susceptible hosts interacting with the vector. One of the main vectors for viruses infecting humans are mosquitoes. These insects transmit dangerous arboviruses, whose transmission can be enhanced or suppressed by the mosquitoes’ microbiome (54). The mosquito Aedes aegypti is a vector for multiple viruses, including the flaviviruses dengue virus (DENV) and Zika virus (ZIKV). Carlson et al. (55) used Bacillus thuringiensis or Enterobacter ludwigii to feed A. aegypti in three combinations: exposing the mosquitoes to the bacteria only during larval stage, only as adults, or in both stages. They found that exposure to B. thuringiensis did not affect either the DENV or the ZIKV infection intensity. In contrast, mosquitoes exposed to *E. ludwigii* only during their larval stage showed a reduced intensity of infection by DENV. For ZIKV, the opposite was observed: mosquitoes exposed to this enterobacterium in their larval and adult stages showed increased ZIKV infection. A recent study observed that the exposure of larvae to different bacteria influences adult competence for virus transmission (56). The effect of the microbiome on virus transmission may be dependent on the interacting species. For example, hosting the protozoan parasite Ascogregarina culicis does not alter the DENV dissemination rate by A. aegypti (57). Studying mice with an altered microbiome, Winkler et al. (48) found that providing mice with the bacterium *C. scindens* not only limited the CHIKV infection but also prevented the transmission to a mosquito vector.

The microbial mitigation of virus transmission has led to the development of microbiome-based approaches to reduce the impact of vector-borne viruses. The bacterium *W. pipientis* is now being artificially and stably introduced into A. aegypti to reduce the transmission of the several viruses vectored by the mosquito. This intervention will reduce the outbreak of, e.g., DENV or ZIKV (58). The microbiome approach may also be effective in preventing the spread of plant diseases. The planthopper Nilaparvata lugens is a pest that transmits rice ragged stunt virus (RRSV) to different cultivars, causing catastrophic crop losses. Gong et al. (59) introduced *Wolbachia* strain *w*Stri into *N. lugens*, resulting in reduced infection and transmission of RRSV and less severe symptoms in infected plants.

### Plants.

Plant viruses have a tremendous impact on wild ecosystem and agro-ecosystem stability and function, causing major economic losses and endangering the food security of human populations (60). Unsurprisingly, the plant’s microbiome also plays a key role in plant health (61, 62). Depending on its location, the plant microbiome varies, and it can be classified as rhizosphere (underground plant’s immediate surroundings), phyllosphere (aerial plant’s immediate surroundings) and endosphere (within the plant tissues) (63). Upon pathogen or insect attack, plants are able to recruit protective microorganisms and enhance microbial activity to suppress pathogens in the rhizosphere (64). The plant microbiome can expand its immunity, acting as a defensive layer against pathogens: the microbiome can mitigate the impact of pathogens thanks to direct interactions with them or by priming the plant’s defensive response (65).

The impact of the plant microbiome on infectious disease has been extensively studied for nonviral diseases (66, 67). Concerning viruses, Safari et al. (68) showed that jalapeño pepper plants that were asymptomatically and persistently infected with pepper cryptic virus 1 were less attractive to aphids. As aphids are usually vectors of pathogenic plant viruses, the reduction of interactions with them minimizes the risk of the plant infections. Bonanomi et al. (69) described an association of the abundance of some soil microbiota and infection with tomato spotted wilt virus (TSWV). In particular, there was a negative correlation between the abundance of *Acremonium* fungi and bacteria (*Actinobacteria* spp., Pseudomonas spp., and *Agrobacterium* spp.) and TSWV rates of infection and severity of disease.

There is a promising future in microbiome-based interventions to improve plants’ defense against virus. This possibility has been shown for other pathogens. Inoculation of germinating plants with a native bacterial consortium significantly attenuates the plants’ mortality against bacterial and fungal pathogens (70). Transplantation of rhizosphere microbiota from resistant plants suppressed fungal disease symptoms in susceptible plants (71).

### Species conservation.

Species face multiple threats that can reduce their population size. One of these threats is infectious disease, which is possibly a main factor in extinction risk (72). Therefore, conveniently modifying the microbiome of an endangered species could help to preserve it by enhancing its health. There are calls for raising awareness of the importance of the microbiome in the conservation of species (73). For example, canine distemper virus (CDV) has caused a decline in the population of many wildlife species. Zhao et al. (74) studied CDV-infected and healthy giant pandas and observed that CDV-infected individuals had their gut bacterial composition strongly altered.

In some cases, a pathogenic virus is not the main cause of a species decline, but it is a factor contributing to it. This is the case of the worldwide population of Apis melifera, whose decline is mainly driven by habitat loss, pesticides, and several pathogens (75, 76), including deformed wing virus. As the microbiome shapes the innate immunity of the bees (77), the bees’ microbe composition could be modulated to mitigate viral infections (78, 79) and therefore reduce the decline of the population caused by viral diseases.

## MICROBIOME-MEDIATED ENHANCEMENT OF VIRAL INFECTIONS

The host microbiome can enhance pathogen infection by modifying the within-host environment interacting with the pathogen or driving the pathogen (80). In the case of pathogenic viruses, some microbiomes are associated with higher susceptibility to infection. McClelland et al. (81) found a correlation between higher viral infection susceptibility and the microbiome: women that had an increased risk of human immunodeficiency virus type 1 (HIV-1) acquisition had microbiomes with a high bacterial diversity and dominated by the presence of *Mycoplasma* spp., Prevotella bivia, Prevotella melaninogenica, Sneathia sanguinegens, and Veillonella montpellierensis.

In some cases, a certain microbiome can be essential for a virus to infect its host. Jones et al. (82) showed that human norovirus needs *Enterobacteria* spp. to successfully infect B cells, as this bacterium aids in the attachment of the virus to the host cell. Norovirus infection in mice requires the microbiota to be persistent (83): mice with a depleted microbiome prevented persistent norovirus infection. This happens as the microbiota suppresses IFN-λ expression, enabling efficient viral persistence. Jones et al. (82) also studied the effect of *Enterobacteria* spp. on murine norovirus, finding that for this virus, the presence of the bacterium is not a requirement for successful host infection. However, the microbiome does have a positive effect on the murine norovirus, as this virus shows a reduced replication in axenic mice. This exemplifies (i) how the microbiome may have different effects on virus infection, depending on the host, and (ii) how the microbiome may not be necessary for an infection to happen, although it can affect the interaction of the virus with the host: the microbiome may regulate important phenotypes of the pathogen in terms of the degree of virus replication or transmission. There are other pathosystems in which an effect of the microbiome on virus replication and transmission was observed. Axenic mice did not transmit the mouse mammary tumor virus (MMTV) to their offspring. Reconstitution of the mouse bacterial community restored MMTV transmission. This seems to happen as MMTV binds bacterial lipopolysaccharides to trigger Toll-like receptor 4, inducing an immune evasion pathway by producing inhibitory cytokine IL-10 (84, 85). Gulraiz et al. (86) showed that, in human bronchial epithelial cells, Haemophilus influenzae increases the expression of a receptor used by human rhinoviruses. This results in an enhanced virus replication and inflammatory response to RSV. In the case of poliovirus (PV), the virions’ stability and capacity to attach to host cells are enhanced when the virus binds to bacterial surface polysaccharides. This increase in stability and receptor affinity suggests that the microbiome may also increase PV replication and transmission (87, 88). Likewise, the interaction with bacterial envelope components also enhances reovirus thermostability. The enhanced virion tolerance to temperature being due to the interaction of intermediate reovirus particle with bacterial lipopolysaccharides and peptidoglycans (89).

In some cases, the microbiome could also increase the symptomatology or the consequences of viral infection. For example, human papillomavirus (HPV) can cause cervical intraepithelial neoplasia, which could lead to cervical cancer. Oh et al. (90) found a positive association between cervical intraepithelial neoplasia incidence in people infected with HPV and a high prevalence of Atopobium vaginae in the cervical microbiome. De Steenhuijsen Piters et al. (91) found an association between nasopharyngeal microbiota dominated by H. influenzae and Streptococcus spp. and enhanced disease severity caused by RSV. Ramani et al. (92) found an association between the relative abundance of Enterobacter spp./Klebsiella spp. in mothers’ milk and human rotavirus (HRV)-induced gastrointestinal symptoms in newborns. Axenic chickens infected with Marek’s disease virus had more severe disease (93). Similar enhancements can happen in plants, as plants persistently infected with Southern tomato virus develop stronger symptoms when infected with pathogenic viruses, such as cucumber mosaic and pepino mosaic viruses (94).

## MICROBIOME INTERACTION WITH ANTIVIRAL THERAPEUTICS

To treat or prevent viral infections, we mainly rely on two interventions: antiviral drugs and vaccines. The effect of antiviral drugs could require the microbiota or be enhanced using microbiome interventions, as the microbiome can affect the degree of efficacy and toxicity of the drug along with drug metabolization (95–98). Some studies point to a possible microbiome-dependent action for some antivirals. For example, women’s vaginal microbiome dysbiosis reduces the efficacy of an antiviral drug against HIV-1 (99). In mice, the peptides Serp-1 and S-7 reduce the pulmonary pathology caused by MHV-68. This disease severity mitigation is partially decreased in microbiome-depleted mice (48). The drug-microbiome interaction flows both ways, since drugs can alter the microbiome’s composition (100).

Despite the usefulness of vaccines to fight viral infections, the efficacy of a vaccine is highly variable among individuals within a population. The variability in the protective immunity conferred by a vaccine is caused by many factors. One of these factors seems to be the microbiome differences among individuals (101, 102). A growing number of studies suggest that the microbiome can modulate immune responses induced by vaccines (103, 104). Huda et al. (105) studied infants’ microbiomes to evaluate how their microbiome composition influenced the response to oral PV and hepatitis B virus vaccines. They found that *Actinobacteria* species prevalence may increase the vaccine response, while *Enterobacteriales*, *Pseudomonadales*, and *Clostridiales* were associated with lower response to the aforementioned vaccines. Hagan et al. (106) showed that differences in the microbiome can alter the responses of humans vaccinated against IAV. In this study, subjects vaccinated with the trivalent inactivated IAV had a reduced IgG1 and IgA response. This reduction was significant only in subjects with low baseline levels of neutralizing antibodies who were vaccinated against the H1N1 strain. In another study, Fix et al. (107) found that the infants who responded to HRV vaccination tended to have higher abundance of *Proteobacteria* spp. and *Eggerthella* spp., but the differences found in this study were not statistically significant. Therefore, the impact of the microbiome on the HRV vaccination can be limited or influenced by other factors. Other associations have been established between the microbiome of an organism and the organism’s response to vaccines. Kandasamy et al. (108) evaluated the effect of an attenuated HRV vaccine on piglets colonized with Lactobacillus rhamnosus and Bifidobacterium animalis. After being challenged with HRV, the cocolonized animals showed an enhanced intestinal HRV IgA antibody titer and a decrease in reduced HRV shedding. Sui et al. (109) pointed to a positive correlation between the microbiome composition and the immunization induced by the SIV vaccine. A descriptive study of rhesus macaques’ response to HIV-1 vaccine depending on their microbiome found that the macaques’ rectal microbiome composition correlated with the antibody response generated by the HIV-1 vaccine: *Lactobacillus* species abundance had a strong association with higher IgA levels (110). Musich et al. (111) vaccinated rhesus macaques and then challenged them with SIV. Their results suggest the impact of the rectal microbiome on the immune response induced by vaccine varied between males and females. Furthermore, they observed a correlation between the presence of *Proteobacteriales*, *Epsilonproteobacteriales*, and *Campylobacterales* and a decrease in the peak viral load in vaccinated females. Importantly, the vaccine-microbiome interaction occurred both ways as the immunization also induced changes in the composition of the macaque’s rectal microbiome. In pigs, the microbiome was found to be associated with PRRSV vaccine efficacy in animals challenged with PRRSV and PCV2. In addition, the microbiome composition after vaccination was a determinant of the animal growth rate (112).

Lynn et al. (113) studied the effect of antibiotic-driven dysregulation of the gut microbiota in mice. They observed that infants with dysregulated microbiome had an impairment in antibody responses to five different adjuvanted and live vaccines. The antibody response was normal when vaccines were applied to dysbiotic adults. Harris et al. (114) studied the effect of the microbiome by using antibiotics to deplete it. They established three groups of humans: a placebo group, a narrow-spectrum antibiotic group, and a broad-spectrum antibiotic group. After 36 h, the three groups received the pneumococcus, tetanus, and HRV vaccines. The immune response of the pneumococcus and tetanus vaccines was not altered by the antibiotic treatment. For the HRV vaccine, they observed that the group treated with the narrow-spectrum antibiotic had a higher IgA boosting. This increase in the secondary immune response correlates with an expansion of the *Proteobacteria* spp. in the microbiome. These results confirm that the composition of the microbiome correlates with the response to the HRV vaccine, as previously shown (115, 116). Chickens vaccinated with avian influenza virus showed an increased response to the vaccine if the individuals were previously supplemented with five *Lactobacillus* spp. In comparation with chickens treated with antibiotics, the chickens with a modified microbiome had higher IgM, IgG, and IFN-γ levels (117). There may be multiple vaccines for which the microbiome has no influence. Oh et al. (118) observed an interaction between the microbiome and the trivalent inactivated IAV vaccine. In their study, axenic mice had impaired plasma cell and antibody responses to the IAV vaccination. However, the authors did not observe an effect of antibiotics on the antibody response generated by tetanus-diphtheria-pertussis vaccine and live attenuated yellow fever vaccine.

In summary, the effect of certain therapeutics can be altered by the microbiome. This effect seems to be specific to the microbiota and the therapy. Therefore, in some cases, microbiome interventions could be useful to maximize the viral immunity provided by therapeutic interventions.

## FUTURE PERSPECTIVES

The impact that a given microbiome has on pathogenic viruses may depend on the environment, host and virus genetics, and other factors. However, there is enough evidence to affirm that some microbiomes can enhance or mitigate viral infections in hosts from different life kingdoms. As this field expands, more microbes that have an effect on viral infections will be discovered.

Future research will shed light on the mechanism behind the interaction between microbes and pathogenic viruses and the specificity of the interaction. It will be important to study not only the impact of specific microbes alone, but also the interaction of viruses with complex microbial communities. The variation in the composition of microbiomes may result in epistatic effects on viral infections, whereas microbes that do not have any effect on pathogenic viruses by themselves may be able to mitigate viruses in the presence of other microbes. It will be necessary to characterize the universality of the impact of specific microbiota: (i) the effect of a specific microbiome on one virus might not be the same for other virus strain or species, and (ii) the relationship between the microbiome and virus infections could be unique at the host species level (119). Furthermore, future studies involving microbiota manipulation should corroborate associations found in descriptive studies. Altogether, this very much needed research will expand the knowledge about the beneficial microbes, their means of action, and the conditions under which this mitigation occurs. This information is key to engineering microbial communities aimed at reducing the impact of pathogenic viruses. Therapeutic approaches to fight viral infections should include the modulation of the microbiome (120). As an example of the power of these interventions, the reconstitution of the wild-like mouse microbiome into laboratory mice improved the outcome of viral infection of laboratory mice: microbiome-reconstituted mice survived otherwise lethal infection with IAV (121). There are interventions already under way aiming to restore the aged microbiome to boost host immunity (122) or improvements in soil management to drive microbiome composition and therefore reduce the incidence of plant viruses (69). The modulation of the microbiome may also help to prevent secondary infections, as viral infections disrupt microbiome composition, and this dysbiosis facilitates the infection of other pathogens (123). The implementation of microbiome-based interventions will have a higher impact when the microbiome-mediated mitigation is maintained over time. Kloock et al. (124) showed that C. elegans maintained a microbe that conferred protection against bacterial infection even when the bacterial pathogen was absent. New approaches can facilitate the formation of dynamically stable and ecologically resilient microbial communities (125).

Finally, the microbiome could be used not only for altering the viral infection outcome, but also for driving the evolution of the virus. Ford et al. (126) showed that the presence of Enterococcus faecalis in the host microbiome drove the evolution of a bacterial pathogen toward reduced virulence. Following the idea of redirecting virus evolution toward less pathogenic strains (127), the microbiome could be used to drive the virus’s evolution for the host’s benefit: it could be possible to evolve the host’s microbes in order to get them to mitigate viral infections. It has been experimentally shown that microbes can evolve into a beneficial relationship with their host when facing a pathogen (128) or adverse environments (129). When driving the evolution of the microbiota, it is necessary to explore the impact of the evolved microbes in their host. In order to implement this approach, it is also essential to ensure that the evolved microbes do not disrupt the normal function of the microbiome. As an example, a bacterial strain experimentally evolved to protect its host from bacterial infection did not have a significant impact on the host’s microbiome (130). However, other evolved microbes may have a negative impact, and each case should be evaluated individually. Altogether, this future research will allow us to implement the best and safest approach in each situation to exploit microbiome-virus interactions.

## CONCLUDING REMARKS

The microbiome can play a fundamental role in viral infections. The host’s microbiome may determine the success of a virus infection in an organism and/or the severity of the viral disease. The research on the microbiome-virus interactions is promising: the advances in the near future should give us insights into the nature and mechanisms behind the microbiome’s influence on viral infections. Microbiome-virus research is developing across various fields and organisms. This research will aid the development of interventions that reduce the viruses’ impact, which could be applied to different hosts. These microbiome-based interventions would contribute to the establishment of an integrated health approach to face pathogenic viruses and reduce the impact of diseases.
